# Graded Hypercapnia-Calibrated BOLD: Beyond the Iso-metabolic Hypercapnic Assumption

**DOI:** 10.3389/fnins.2017.00276

**Published:** 2017-05-18

**Authors:** Ian D. Driver, Richard G. Wise, Kevin Murphy

**Affiliations:** ^1^Cardiff University Brain Research Imaging Centre, School of Psychology, Cardiff UniversityCardiff, United Kingdom; ^2^School of Physics and Astronomy, Cardiff UniversityCardiff, United Kingdom

**Keywords:** fMRI, calibrated BOLD, CMRO_2_, hypercapnia, arterial spin labeling

## Abstract

Calibrated BOLD is a promising technique that overcomes the sensitivity of conventional fMRI to the cerebrovascular state; measuring either the basal level, or the task-induced response of cerebral metabolic rate of oxygen consumption (CMRO_2_). The calibrated BOLD method is susceptible to errors in the measurement of the calibration parameter *M*, the theoretical BOLD signal change that would occur if all deoxygenated hemoglobin were removed. The original and most popular method for measuring *M* uses hypercapnia (an increase in arterial CO_2_), making the assumption that it does not affect CMRO_2_. This assumption has since been challenged and recent studies have used a corrective term, based on literature values of a reduction in basal CMRO_2_ with hypercapnia. This is not ideal, as this value may vary across subjects and regions of the brain, and will depend on the level of hypercapnia achieved. Here we propose a new approach, using a graded hypercapnia design and the assumption that CMRO_2_ changes linearly with hypercapnia level, such that we can measure *M* without assuming prior knowledge of the scale of CMRO_2_ change. Through use of a graded hypercapnia gas challenge, we are able to remove the bias caused by a reduction in basal CMRO_2_ during hypercapnia, whilst simultaneously calculating the dose-wise CMRO_2_ change with hypercapnia. When compared with assuming no change in CMRO_2_, this approach resulted in significantly lower *M*-values in both visual and motor cortices, arising from significant dose-dependent hypercapnia reductions in basal CMRO_2_ of 1.5 ± 0.6%/mmHg (visual) and 1.8 ± 0.7%/mmHg (motor), where mmHg is the unit change in end-tidal CO_2_ level. Variability in the basal CMRO_2_ response to hypercapnia, due to experimental differences and inter-subject variability, is accounted for in this approach, unlike previous correction approaches, which use literature values. By incorporating measurement of, and correction for, the reduction in basal CMRO_2_ during hypercapnia in the measurement of *M*-values, application of our approach will correct for an overestimation in both CMRO_2_ task-response values and absolute CMRO_2_.

## Introduction

Blood oxygenation level dependent (BOLD) functional MRI (fMRI) signal contrast is widely used as a surrogate measure of underlying neuronal activity (Kwong et al., 1992; Ogawa et al., 1992). The BOLD signal is dependent on the concentration of deoxygenated hemoglobin in blood, which is modulated by changes in cerebral blood flow (CBF), cerebral blood volume (CBV), and cerebral metabolic rate of oxygen consumption (CMRO_2_). Significant inter-region and inter-subject variability in the BOLD response arises due to vascular factors (Chiarelli et al., 2007a; Lu et al., 2008), which could confound interpretation of the underlying neuronal activity from BOLD results. The technique termed calibrated BOLD was developed to address this variability (Davis et al., 1998; Hoge et al., 1999). A calibration parameter (*M*), defined as the theoretical BOLD signal change that would occur if all deoxygenated hemoglobin were removed, incorporates basal hemodynamic parameters, along with parameters that are dependent on the specific MRI acquisition scheme. This calibration parameter allows for calculation of either absolute CMRO_2_ (Bulte et al., 2012; Gauthier and Hoge, 2012; Wise et al., 2013), or the task-dependent relative change in CMRO_2_ (Davis et al., 1998; Hoge et al., 1999). CMRO_2_ directly reflects tissue metabolism, so is widely considered a more direct measurement of underlying neuronal activity than the cerebrovascular CBF and CBV responses. However, this approach is sensitive to errors in the measurement of *M* (Hoge et al., 1999; Chiarelli et al., 2007b).

The original and most popular method for calculating *M* uses hypercapnia (an increase in arterial CO_2_). Typically, hypercapnia is presented through inhalation of gas mixtures including CO_2_, or through reductions in breathing depth and/or pace (e.g., breath hold). The cerebral vasculature is sensitive to changes in partial pressure of carbon dioxide dissolved in arterial blood (PaCO_2_), such that hypercapnia is a potent vasodilator, causing increases in CBF and CBV (Kety and Schmidt, 1948; Ito et al., 2003; Noth et al., 2006; Chen and Pike, 2010b; Ho et al., 2011). For calibrated BOLD, hypercapnia is assumed to be a purely vascular stimulus, providing a change in CBF and CBV without a change in CMRO_2_ (Davis et al., 1998). However, this iso-metabolic assumption is the subject of controversy (Yablonskiy, 2011), with literature indicating increases, decreases and no change in basal CMRO_2_ with hypercapnia (Kety and Schmidt, 1948; Kliefoth et al., 1979; Rhodes et al., 1981; Hoffman et al., 1982; Horvath et al., 1994; Jones et al., 2005; Sicard and Duong, 2005; Chen and Pike, 2010a; Jain et al., 2011; Xu et al., 2011). Recent human MRI studies (Chen and Pike, 2010a; Jain et al., 2011; Xu et al., 2011) have shown mixed results, calculating CMRO_2_ using the Fick principle to combine CBF (phase contrast MRI) and OEF (either T_2_- or susceptibility-based venous blood oxygenation measurements). Xu et al. found a significant 13.4 ± 2.3% decrease with a 5% CO_2_ hypercapnic challenge, whilst Chen and Pike and Jain et al. found no significant change in basal CMRO_2_ with hypercapnia. Whilst these contrasting findings may arise due to methodological differences, any CMRO_2_ change with hypercapnia appears to be on the order of the measurement accuracy of these methods. Despite this limited sensitivity, recent calibrated BOLD studies have begun to correct for an assumed reduction in basal CMRO_2_ with hypercapnia (Bulte et al., 2012), based on these literature values (Xu et al., 2011). An appropriate choice of CMRO_2_ response (or lack thereof) to hypercapnia is important for calibrated BOLD experiments due to the sensitivity of the method to propagation of errors in M through to the endpoint CMRO_2_ calculation (Hoge et al., 1999; Chiarelli et al., 2007b; Blockley et al., 2015).

With measurement sensitivity on the order of the changes observed, current MR techniques are unsuited to fully characterize the relationship between PaCO_2_ and CMRO_2_. However, electrophysiological measurements appear to have more sensitivity to investigate the dynamic range of mild hypercapnia PaCO_2_ values. Studies in both non-human primates and, more recently, in humans show reductions in spontaneous neuronal oscillatory power with hypercapnia (Jones et al., 2005; Zappe et al., 2008; Hall et al., 2011; Xu et al., 2011). We have recently observed a linear relationship between PaCO_2_ and spontaneous neuronal oscillatory power (Driver et al., 2016). Whilst there is currently no direct relationship established between CMRO_2_ and neuronal oscillatory power, they may share similar underlying neurochemical mechanisms for their responses to hypercapnia, specifically extracellular pH changes modulating ATP channels (Dulla et al., 2005). Therefore, in the following paragraphs, we explore the addition to the calibrated BOLD technique of an assumption of a linear relationship between hypercapnia level and CMRO_2_.

In this work, we present a new approach, relaxing the iso-metabolic hypercapnia assumption in the calibrated BOLD technique. By acquiring multiple levels of hypercapnia, *M* and the dose-wise CMRO_2_ response to hypercapnia are solved for as two unknowns in a set of simultaneous equations (one equation for each hypercapnia level). We apply this approach to data presented previously (Murphy et al., 2013), measuring both the calibration parameter *M* and the dose-wise CMRO_2_ response to a graded hypercapnia challenge.

## Materials and methods

### Theory

The calibrated BOLD equation, as proposed by Davis et al. (1998) and elegantly restated by Hoge et al. (1999) can be used to model the BOLD signal change during hypercapnia:

(1) $$\begin{array}{c} \frac{\Delta BOLD_{HC}}{BOLD_{0}}=M\left[1-{\left(\frac{CBF_{HC}}{CBF_{0}}\right)}^{\alpha -\beta }\cdot {\left(\frac{CMRO_{2,HC}}{CMRO_{2,0}}\right)}^{\beta }\right] \end{array}$$

Where *M* is the calibration parameter, Δ denotes the difference between the current state and baseline state, subscripts *HC* and *0* denote hypercapnia and baseline (normocapnia) conditions, respectively. The exponent α describes an assumed coupling relationship between CBF and CBV (Grubb et al., 1974; Chen and Pike, 2010b), whilst β is a power-law relationship between venous blood oxygenation and transverse relaxation rate (Ogawa et al., 1993; Boxerman et al., 1995; Driver et al., 2010; Croal et al., 2017). If the iso-metabolic assumption were used, the CMRO_2_ term would reduce to 1, with numerator and denominator being equal. *M* could then be calculated from Equation (1) using measured values for the relative changes in BOLD and CBF due to hypercapnia.

We propose to remove the iso-metabolic assumption, such that the CMRO_2_ term becomes an unknown parameter to be solved alongside *M*. To do this, we measure BOLD and CBF responses to two levels of hypercapnia, setting up two versions of Equation (1). Since the CMRO_2_ term may change between the two equations, we assume a linear relationship between CMRO_2_ and hypercapnia level. A new parameter κ is defined as the dose-wise fractional CMRO_2_ change to a unit (1 mmHg) change in end-tidal partial pressure of carbon dioxide (P_ET_CO_2_), a surrogate measure for PaCO_2_.

(2) $$\begin{array}{c} \frac{CMRO_{2,HC}}{CMRO_{2,0}}=1+\kappa \cdot \Delta P_{ET}CO_{2} \end{array}$$

Substituting Equation 2 into Equation 1:

(3) $$\frac{\Delta BOLD_{HC}}{BOLD_{0}}=M\left[1-{\left(\frac{CBF_{HC}}{CBF_{0}}\right)}^{\alpha -\beta }\cdot {\left(1+\kappa \cdot \Delta P_{ET}CO_{2}\right)}^{\beta }\right]$$

Therefore, with P_ET_CO_2_ measured by sampling exhaled gas, *M* and κ are two unknowns, which can be solved for using two equations, one for each hypercapnia level.

### Data acquisition

Fifteen subjects (7M/8F, age range 21–36 years) participated in 2 sessions in which scans were acquired using a 3 T whole body MRI system (GE Excite HDx, Milwaukee, WI, USA) with an eight-channel receive coil. The School of Psychology, Cardiff University Ethics Committee approved this study and subjects gave written informed consent prior to participating.

Data were acquired using a pulsed arterial spin labeling (ASL) proximal inversion and control for off-resonance effects (PICORE), quantitative imaging of perfusion using a single subtraction (PICORE QUIPSS II) (Wong et al., 1998) imaging sequence. This sequence used a dual-echo gradient echo readout (Liu et al., 2002) and spiral-out k-space acquisition [Glover, 1999; 490 repetitions (image volumes), TE1 = 3.3 ms TE2 = 29 ms, TR = 2,200 ms, flip angle 90°, FOV 22 cm, matrix 64 × 64, 12 slices of 7 mm thickness with an inter-slice gap of 1 mm, TI1 = 600 ms, TI2 = 1,500 ms for the most proximal slice, 10 cm inversion slab thickness, adiabatic hyperbolic secant inversion pulse, 10 mm gap between labeling slab and bottom slice, 10 cm QUIPSS II saturation band thickness]. Additionally, whole brain T_1_-weighted structural scan (fast spoiled gradient recalled echo, 1 × 1 × 1 mm voxels, TI/TR/TE = 450/7.8/3 ms) was acquired for segmentation of gray matter.

Participants were presented with hypercapnia levels of +4 and +8 mmHg ΔP_ET_CO_2_ above their normal resting level. End-tidal CO_2_ levels were changed at 2-min intervals between baseline, +4 and +8 mmHg values, in a randomized order. This provided three 2-min blocks for each condition, across the 18 min ASL scan. Gas mixtures were delivered to the subject through a tight-fitting face-mask (Quadralite, Intersurgical, Wokingham, Berkshire, UK). Flow rates of two gas mixtures, namely medical air (21% O_2_, 79% N_2_) and a 5% CO_2_ mixture (5% CO_2_, 20% O_2_, 75% N_2_), were manually adjusted to provide an inspired gas mixture of 30 L/min. The respiratory circuit included a reservoir on the expired limb to permit re-breathing in the event that the instantaneous inspiratory rate exceeded 30 L/min. Expired gas concentrations were sampled from the face-mask and P_ET_CO_2_ and P_ET_O_2_ (end-tidal pO_2_) were measured using rapidly responding gas analyzers (AEI Technologies, Pittsburgh, PA, USA). A manual feedback procedure was used to reach each hypercapnia level, whereby the respective flow rates of medical air and the 5% CO_2_ mixture were adjusted to reach the P_ET_CO_2_ target.

A combined visual and motor task was simultaneously performed, consisting of blocks of 8 Hz flashing checkboard and right-handed self-paced finger tapping with a range of block durations between 20 and 30 s. These blocks were interspersed with 20–30 s blocks of rest. The range in task and rest block durations were chosen so that the visual, motor and CO_2_ tasks had minimal correlation with each other. The task data was used to define primary visual and motor cortex ROIs.

### Data analysis

CBF time series were calculated from the first echo by separating tag and control time series, interpolating to the TR and subtracting. A similar procedure using averaging rather than subtraction yielded BOLD time series from the second echo. R_2_^*^ was also calculated by performing an exponential fit across the two echo times, separately for tag and control time series, then combining tag and control R_2_^*^ values by surround averaging. A gray matter (GM) ROI was calculated for each subject by segmenting their anatomical image into three tissue types (gray matter, white matter and cerebrospinal fluid) using FSL's *fast*. The GM map was resampled to the functional data resolution. Visual and motor ROIs were calculated by including CO_2_, visual and motor timings in a voxel-wise GLM for both the BOLD and CBF data. The results for each subject were transformed into MNI space and a voxel-wise *t*-test against 0 across subject was performed. The *t*-test maps were FDR thresholded at *p* = 0.05. The resulting BOLD and CBF activation maps were transformed back into individual subject space. The visual and motor ROIs were then calculated for each subject by taking an *intersection* map between the BOLD activation map, the CBF activation map and the individual GM ROI. Therefore, a voxel was only included in the motor ROI if it significantly responded to the motor task in both the BOLD and CBF data and was present in the individual's GM mask. A similar procedure was used for the visual ROI. Once the ROIs were defined, the BOLD and CBF time series were averaged over the visual, motor, and GM ROIs, then linear detrending was performed using baseline periods, before averaging across sessions for each subject.

BOLD and CBF responses to each hypercapnia level (relative to baseline) were input into +4 and +8 mmHg versions of Equation (3), then these two equations were solved simultaneously for *M* and κ, using a two-parameter non-linear fitting routine (*lsqcurvefit*, Matlab, The MathWorks, Natick, USA). Subjects that reached the boundary conditions of the non-linear fitting routine were removed from further analysis (boundary conditions 1 <M<20%; −5 < κ < +5%/mmHg). For comparison with the iso-metabolic assumption, the same two equations as above (+4 and +8 mmHg versions of Equation 3) were solved simultaneously using a one-parameter fit, to calculate M whilst fixing κ = 0.

Optimized values of α = 0.14 and β = 0.91 were used (Griffeth and Buxton, 2011), hereafter referred to as the *empirically derived* α*/*β *pairing*. To ensure that our findings were not biased by choice of this α/β pairing, we also repeated the non-linear fitting with the following two alternative α/β pairings that have been used previously for calibrated BOLD experiments at 3 T. Values of α = 0.2 and β = 1.3 have been used at 3 T (Bulte et al., 2012), hereafter referred to as the *3 T specific* α*/*β *pairing*. Finally, a simplified model has been proposed recently, substituting α/β for a single parameter θ = 0.06 at 3 T (Merola et al., 2016). In this case, this simplified model is equivalent to α = 0.06 and β = 1, hereafter referred to as the *simplified model* α*/*β *pairing*.

## Results

**Figure 1:**
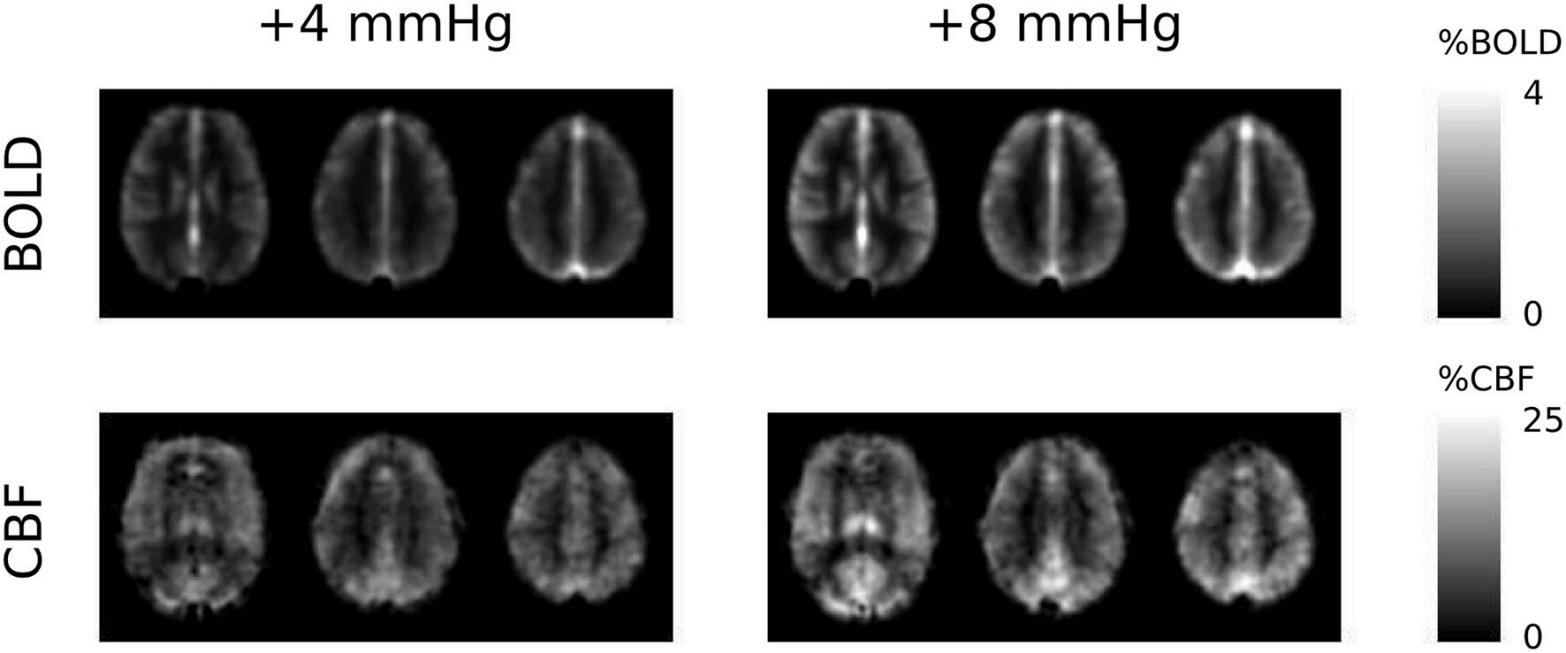
**Group-average maps of BOLD and CBF responses to the +4 and +8 mmHg ΔP_**ET**_CO_**2**_ hypercapnia conditions**. The three slices shown are at the level of MNI coordinate Z = +12, +28, and +42 mm, respectively.

The two levels of hypercapnia resulted in ΔP_ET_CO_2_ increases of 4.8 ± 0.3 and 8.4 ± 0.4 mmHg (mean ± SEM across subjects). BOLD and CBF responses to the two levels of hypercapnia and the respective task (where applicable) are presented for visual, motor cortices and the remaining GM in Table 1. To assess BOLD sensitivity at the 29 ms TE, baseline R_2_^*^ is also reported for each ROI in Table 1. Group-average maps (MNI space) of the BOLD and CBF responses to the two levels of hypercapnia are shown in Figure 1.

**Table 1:** **BOLD and CBF responses (% change from baseline) for the two hypercapnia levels and for the visual and motor tasks (mean ± SEM across subjects)**.

**ROI**	**ΔP_ET_CO_2_(mmHg)**	**%BOLD**	**%CBF**	Baseline R_2_^*^ (s^−1^)
Visual	4.8 ± 0.3	0.9 ± 0.3	13 ± 3	31.3 ± 1.6
	8.4 ± 0.4	1.7 ± 0.3	19 ± 4	
	0 (+ Task)	1.7 ± 0.1	33 ± 3	
Motor	4.8 ± 0.3	0.8 ± 0.1	30 ± 15	22.3 ± 0.7
	8.4 ± 0.4	1.4 ± 0.2	43 ± 16	
	0 (+ Task)	1.3 ± 0.1	73 ± 19	
GM	4.8 ± 0.3	1.0 ± 0.1	13 ± 2	31.7 ± 0.9
	8.4 ± 0.4	1.6 ± 0.1	17 ± 3	

Baseline R_2_

* *values are also presented for each ROI*.

**Figure 2:**
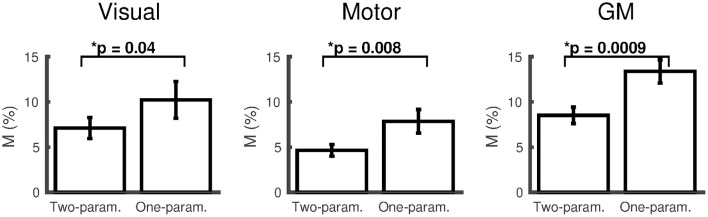
**Comparison of ***M*** (mean ± SEM) calculated using the empirically derived α/β pairing (0.14/0.91) from the two-parameter (ΔCMRO_**2**_ varies linearly with ΔP_**ET**_CO_**2**_) and one-parameter (iso-metabolic) models for subjects that did not reach the boundary conditions for both fits (visual cortex ***N*** = 9; motor cortex ***N*** = 13; remaining GM ***N*** = 14)**. *p < 0.05.

For results presented based on either the two- or one-parameter fits, the number of subjects included after discarding those that reached the boundary conditions are presented in the form (*N* = #/15), where # corresponds to the number of subjects included. The two-parameter fit with the empirically derived α/β pairing (0.14/0.91) gave *M* = 9.6 ± 1.3% (*N* = 14/15) and *M* = 4.7 ± 0.6% (*N* = 13/15), in the visual and motor cortices respectively and *M* = 8.6 ± 0.9% (*N* = 15/15) in the remaining GM. The dose-dependent hypercapnia CMRO_2_ parameter κ = −1.5 ± 0.6%/mmHg, κ = −1.8 ± 0.7%/mmHg and κ = −1.3 ± 0.4%/mmHg showed significant reductions in CMRO_2_ with hypercapnia level (Wilcoxon *p* = 0.04, *p* = 0.03, and *p* = 0.002). The two-parameter fit resulted in significantly lower *M*-values than the one-parameter fit for subjects that did not reach the boundary conditions for both fits [Figure 2; visual *p* = 0.04, (*N* = 9/15); motor *p* = 0.008, (*N* = 13/15); GM *p* = 0.001, (*N* = 14/15)].

**Figure 3:**
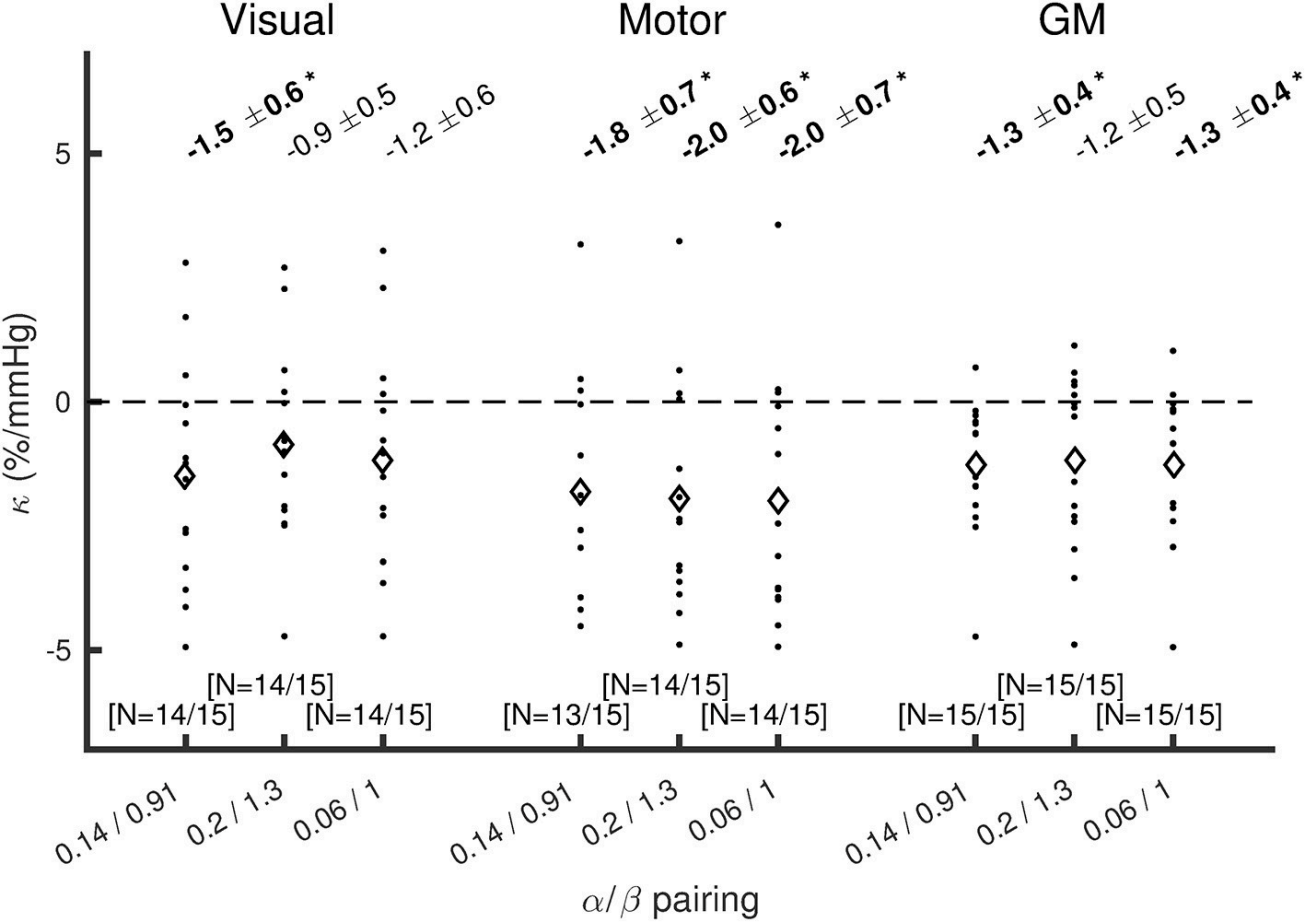
**Plot of κ across subjects for each α/β pairing, for visual cortex, motor cortex, and the remaining GM**. Diamonds show mean κ across subjects. The values presented at the top are mean ± SEM across subjects for κ, with bold text and ^*^indicating p(κ ≠0) < 0.05 (Wilcoxon sign rank test). The numbers of subjects included after discarding those that reached the boundary conditions are presented in the form (*N* = #/15) at the bottom of each plot.

**Figure 4:**
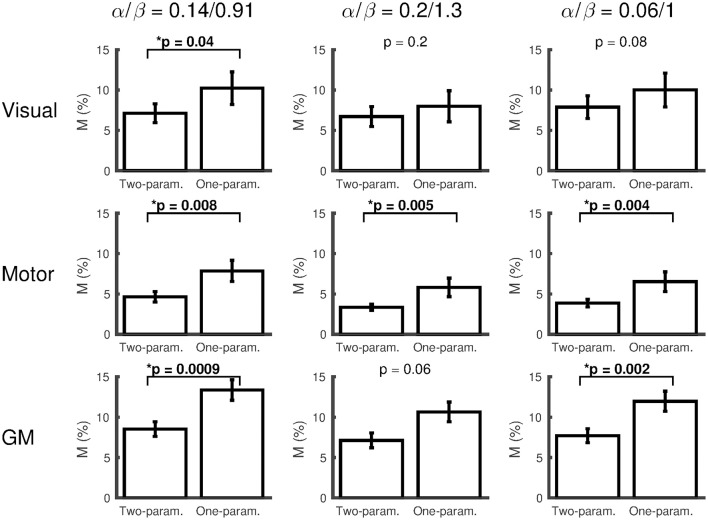
**Plots of ***M*** (mean ± SEM) for each α/β pairing, comparing ***M*** calculated from the two-parameter (ΔCMRO_**2**_ varies linearly with ΔP_**ET**_CO_**2**_) and one-parameter (iso-metabolic) models for subjects that did not reach the boundary conditions for both fits**. The values presented at the top of each plot are Wilcoxon sign rank *p*-values, testing whether *M* differs between two- and one-parameter fits (^*^p < 0.05).

The effect of changing the α/β pairing is assessed in Figures 3, 4. Figure 3 shows a scatter plot of κ across subjects for each α/β pairing and each region of interest, with mean ± SEM across subjects presented above. The group-averaged κ-values remain stable across α/β pairings, indicating a robust decrease in CMRO_2_ during hypercapnia. Figure 4 presents *M* values across α/β pairings. Despite the amplitude of *M* varying with α/β pairing, *M* calculated using the two-parameter fit was consistently lower than that calculated using the one-parameter (iso-metabolic) fit.

## Discussion

Through use of a graded hypercapnia gas challenge, we are able to remove the bias caused by a reduction in basal CMRO_2_ during hypercapnia, whilst simultaneously calculating the dose-wise CMRO_2_ change with hypercapnia. We observed consistently lower *M*-values when calculated from our new approach, compared to those calculated using an iso-metabolic hypercapnia assumption, evidence for a systematic overestimation of *M* when using the iso-metabolic assumption. In terms of studies calculating the relative change in CMRO_2_ to a task, this overestimation in *M* would result in an overestimation in the CMRO_2_ task response (see Equation 1, replacing hypercapnia terms with the equivalent task response terms). In terms of studies investigating absolute CMRO_2_ measurements, the overestimation in *M* would lead to an overestimation of absolute CMRO_2_ (Blockley et al., 2015).

In this study, we define a dose-wise CMRO_2_ response to hypercapnia, κ. Our findings suggest a decrease in CMRO_2_ with hypercapnia in the primary visual and motor cortices, as well as in the remaining GM. The scale of the CMRO_2_ reduction is broadly similar to that observed by Xu et al. (2011), where their 5% CO_2_ challenge resulted in a 13.4% decrease in global CMRO_2_. Based on the reported average ΔP_ET_CO_2_ of 8.7 mmHg, this is equivalent to κ = −1.5%/mmHg. This is within the range of the GM value that we measured of κ = −1.3 ± 0.4%/mmHg. Unlike previous work, which measured the global CMRO_2_ response to hypercapnia, our approach can provide measurements that are localized to specific brain regions. Our initial findings hint at some spatial heterogeneity in the CMRO_2_ response to hypercapnia, with the hypercapnia CMRO_2_ reduction appearing to be greater in the motor than visual cortex. However, this dataset does not have the sensitivity to resolve whether this is a significant difference (see inter-subject variability in Figure 3). Likewise, the paradigm used here is not optimized for voxelwise mapping of *M* and κ, however with a suitably optimized graded hypercapnia paradigm design that enhances voxelwise sensitivity, this approach could be translated to mapping *M* and κ, for application in mapping absolute CMRO_2_. The potential spatial heterogeneity of the CMRO_2_ response to hypercapnia will be investigated in future studies, incorporating a specifically optimized hypercapnia paradigm and a more sophisticated fitting algorithm (Germuska et al., 2016). This approach for mapping the CMRO_2_ response to hypercapnia should be considered in the context of a potential alternative, O-15 PET. There is an extensive literature on mapping CBF and CMRO_2_ using O-15 PET (e.g., Mintun et al., 1984; Ter-Pogossian and Herscovitch, 1985; Kudomi et al., 2013). Whilst there are many studies to use O-15 PET to measure the CBF response to hypercapnia (e.g., Ito et al., 2003), the O-15 PET literature on mapping the CMRO_2_ response to hypercapnia is limited, with no change in CMRO_2_ measured in anesthetized dogs (Rhodes et al., 1981). The steady-state variant of the CMRO_2_ measurement used in that work may also be biased by not accounting for intravascular ^15^O_2_ (Lammertsma and Jones, 1983; Lammertsma et al., 1983; Ter-Pogossian and Herscovitch, 1985). Whilst our method is constrained by the accuracy of the assumptions associated with the calibrated BOLD technique (Hoge et al., 1999; Chiarelli et al., 2007b; Chen and Pike, 2010b; Blockley et al., 2015; Croal et al., 2017), it is non-invasive, not requiring use of radioactive tracers. It also has the potential for finer temporal and spatial resolution than O-15 PET.

The approach we present here requires an assumption as to the form of the relationship between CMRO_2_ and PaCO_2_; in this case, the assumption is of a linear relationship. Beyond the linear relationship, no prior assumption is made as to whether CMRO_2_ increases, decreases or remains constant with hypercapnia. This linear assumption is also implicitly made as part of the iso-metabolic assumption, or when using previous literature values for a CMRO_2_ decrease. The linear relationship between CMRO_2_ and hypercapnia level is based on our recent observations of a linear relationship between hypercapnia level and spontaneous neuronal oscillatory power (Driver et al., 2016). Whilst there is currently no direct relationship established between CMRO_2_ and neuronal oscillatory power, they may share similar underlying neurochemical mechanisms for their responses to hypercapnia, specifically extracellular pH changes modulating ATP channels (Dulla et al., 2005). Even if the relationship includes some non-linearity, bias introduced by a linear correction will be smaller than the bias from no correction.

The constrained non-linear fitting performed here to solve for *M* and κ imposed boundary conditions on these parameters. These boundary conditions 1 < M < 20%; −5 < κ < +5%/mmHg were chosen to be sufficiently broad to include the range of values that would be reasonably expected when averaging across these regions of interest, based on previous literature (Leontiev and Buxton, 2007; Chiarelli et al., 2007a; Mark et al., 2011; Xu et al., 2011). Therefore, where the fitting algorithms returned values that reached these boundary conditions, this is likely to be due to noise in the data, most likely the ASL data, rather than being an actual physiological outlier. It is for this reason that we chose to discard results where the boundary condition was reached, an approach we have taken previously (Murphy et al., 2013; Wise et al., 2013). Out of the 18 versions of the non-linear fitting (three ROIs over three α/β pairings for each of the two- and one-parameter fits), 14 had either one or no subjects reaching the boundary condition. There were 3 occasions where more than two subjects reached the boundary condition, all occurring in the visual ROI for the one-parameter fit, 5 subjects each for the 0.14/0.91 and 0.2/1.3 α/β pairings and 4 subjects for the 0.06/1 α/β pairing. This is consistent with the visual ROI dataset containing more variance than the motor and remaining GM ROIs (see errorbars in Figure 4). Further, a BOLD-weighted second echo time of 29 ms used here has been optimized previously for BOLD contrast, based on GM-averaged R_2_^*^ (Wise et al., 2013; Germuska et al., 2016). The 29 ms echo time is optimal for BOLD contrast for the R_2_^*^ values measured here in visual and GM ROIs (Table 1), however the smaller R_2_^*^ measured in the motor ROI means that BOLD contrast in this ROI is ~10% lower than it would have been if a longer echo time, specifically optimized for the motor ROI, were used. However, since the BOLD contrast is significantly above the noise, this lower BOLD contrast in the motor ROI will have a negligible impact on BOLD sensitivity and will be incorporated into M, not impacting on quantification of CMRO_2_.

In conclusion, we present a new approach to calibrated BOLD, relaxing the iso-metabolic hypercapnia assumption, whilst measuring the dose-wise change in CMRO_2_ due to hypercapnia. This approach can map local CMRO_2_ responses to hypercapnia, so may be suitable for measuring spatial heterogeneity in these responses. This approach may be especially applicable in studies including metabolic pathology, such as diabetes, dementia, and multiple sclerosis, where there may be inter- and/or intra-subject differences in the CMRO_2_ response to hypercapnia. Further, as well as the calibrated BOLD endpoints of measuring task-dependent CMRO_2_, or absolute CMRO_2_, the measurement of the CMRO_2_ response to hypercapnia could become a tool for investigating cerebral metabolic health in its own right.

## Author contributions

ID and KM developed the idea. KM and RW designed the experiment. KM and RW acquired the data. ID and KM performed data analysis. ID, RW, and KM interpreted the results. ID drafted the manuscript. ID, RW, and KM revised the manuscript.

### Conflict of interest statement

The authors declare that the research was conducted in the absence of any commercial or financial relationships that could be construed as a potential conflict of interest.

## Abbreviations

ASL: arterial spin labeling
BOLD: blood oxygenation level dependent (fMRI signal contrast)
CMRO_2_: cerebral metabolic rate of oxygen consumption
CBF: cerebral blood flow
CBV: cerebral blood volume
fMRI: functional magnetic resonance imaging
PaCO_2_: partial pressure of carbon dioxide dissolved in arterial blood
P_ET_CO_2_: end-tidal partial pressure of carbon dioxide
ROI: region of interest.
